# Distinctive pattern of temporal atrophy in patients with frontotemporal dementia and the I383V variant in *TARDBP*

**DOI:** 10.1136/jnnp-2020-325150

**Published:** 2021-01-15

**Authors:** Merel O. Mol, Sebastiaan W.R. Nijmeijer, Jeroen G. J. van Rooij, Resie M. L. van Spaendonk, Yolande A. L. Pijnenburg, Sven J. van der Lee, Rick van Minkelen, Laura Donker Kaat, Annemieke J. M. Rozemuller, Mark R. Janse van Mantgem, Wouter van Rheenen, Michael A. van Es, Jan H. Veldink, Frederic A. M. Hennekam, Meike Vernooij, John C. van Swieten, Petra E. Cohn-Hokke, Harro Seelaar, Elise G.P. Dopper

**Affiliations:** 1 Department of Neurology, Erasmus Medical Center, Rotterdam, The Netherlands; 2 Department of Clinical Genetics, Amsterdam UMC, Location VU University Medical Center, Amsterdam, The Netherlands; 3 Department of Neurology, Alzheimer Center, Amsterdam UMC, Location VU University Medical Center, Amsterdam, The Netherlands; 4 Department of Clinical Genetics, Erasmus Medical Center, Rotterdam, The Netherlands; 5 Department of Pathology, Amsterdam UMC, Location VU University Medical Center, Amsterdam, The Netherlands; 6 Department of Neurology, UMC Utrecht Brain Center, University Medical Center Utrecht, Utrecht, The Netherlands; 7 Department of Clinical Genetics, University Medical Center Utrecht, Utrecht, The Netherlands; 8 Deparment of Radiology and Nuclear Medicine, Erasmus Medical Center, Rotterdam, The Netherlands; 9 Department of Epidemiology, Erasmus Medical Center, Rotterdam, The Netherlands

**Keywords:** frontotemporal dementia, neurogenetics, neuroradiology, neuropathology

## Introduction

Frontotemporal dementia (FTD) and amyotrophic lateral sclerosis (ALS) are closely related disorders, linked pathologically and genetically by the TAR DNA-binding protein-43 (TDP-43). Pathogenic variants in *TARDBP* encoding for TDP-43 have been described less frequently in FTD than in ALS, and clinicopathological studies are scarce.^1^ We previously observed a high frequency of the I383V variant in *TARDBP* in a Dutch cohort of FTD patients.^2^ Here, we provide further evidence for the pathogenicity of this variant and present its clinicopathological characteristics.

## Methods

We ascertained all FTD (n=13) and ALS patients (n=4) with the I383V variant (NM_007375.3: c.1147A>G, p.Ile383Val) in *TARDBP* from three university medical centres in the Netherlands (Amsterdam, Rotterdam and Utrecht), as identified by whole-exome or whole-genome sequencing in either clinical or research setting. Concurrent pathogenic variants in 20 other genes associated with ALS, FTD or other forms of dementia were excluded in all patients.

Brain imaging (CT or MRI) was available for all FTD patients. Quantitative assessment of volume loss across lobar brain regions was performed in those patients with T1-weighted MRI images of sufficient quality (n=5), and compared with a gender-matched/age-matched reference population.

Family histories were classified into adjusted Goldman categories, which were described previously.^2^ Additionally, we performed extensive genealogical research to investigate possible relatedness between the index patients.

Brain autopsy and routine immunohistochemistry was performed for two FTD patients by the Netherlands Brain Bank. One patient (4M) was reported previously as M008015-001.^1^ Detailed information on the genetic, neuroimaging, genealogical and pathological analyses can be found in the1.

10.1136/jnnp-2020-325150.supp1 Supplementary data

## Results and discussion

### The variable clinical phenotype and reduced penetrance of the I383V variant

All 13 FTD patients with the I383V variant in *TARDBP* presented with a combination of behavioural changes and semantic deficits. The diagnoses of semantic variant of primary progressive aphasia (svPPA) are intriguing since this is usually considered a sporadic disorder. One patient (4M) showed additional motor symptoms, but not fulfilling ALS criteria. Of the 4 ALS patients with the I383V variant, 3 had a relatively slow progression with the longest disease duration of 9 years. None of the ALS patients exhibited cognitive or behavioural symptoms. Clinical details are presented in online supplemental tables 1,2.

10.1136/jnnp-2020-325150.supp2 Supplementary data

**Figure 1:**
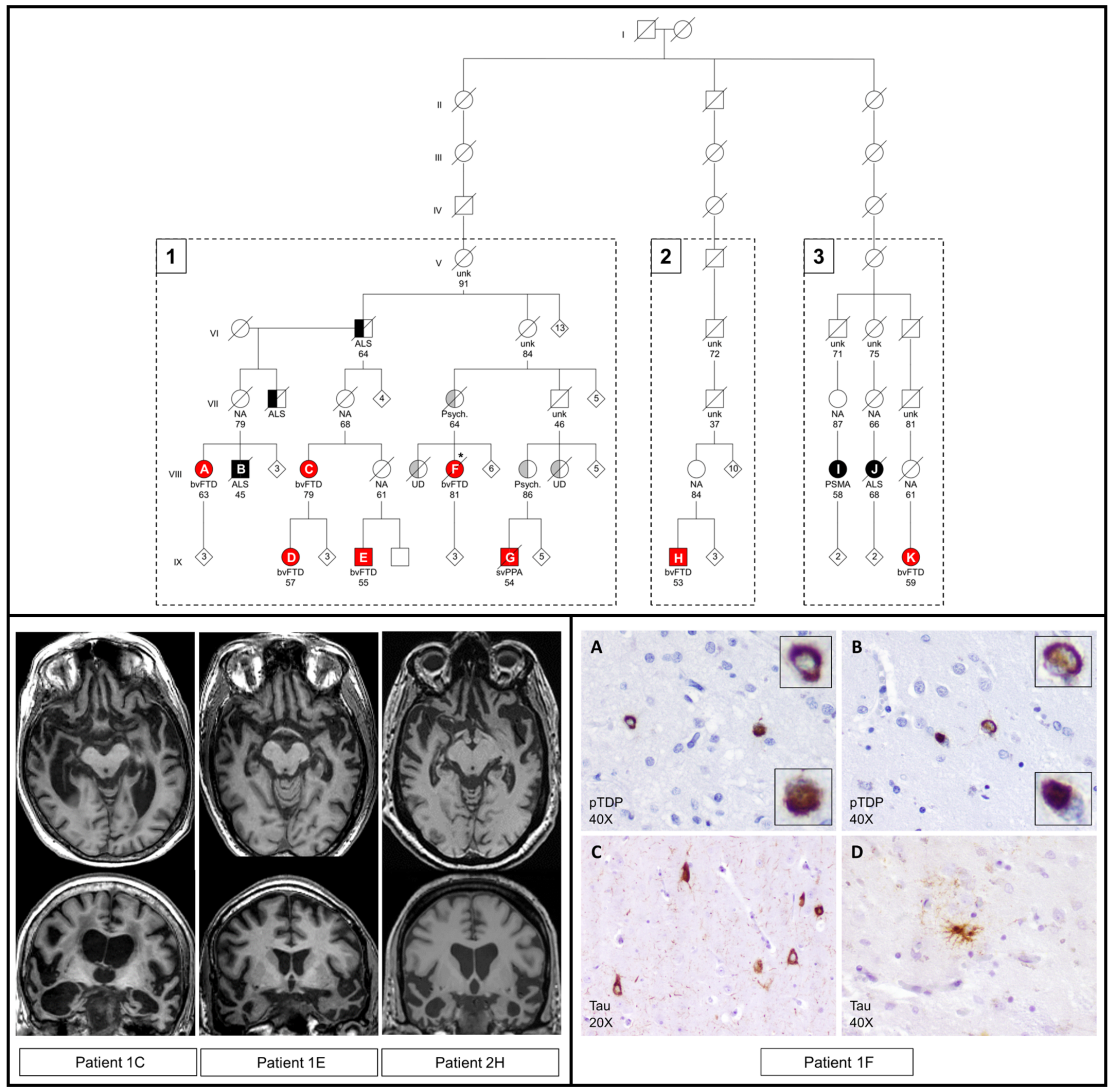
Pedigree of families 1–3 with radiological and pathological features. Upper panel: three families were found to have a common ancestor following genealogical research. These families include 8 FTD patients and 3 ALS patients with a confirmed I383V variant in *TARDBP* (numbered A–K; fully coloured). Half coloured symbols represent patients with a clinical diagnosis without genetic testing. Red: clinical diagnosis of FTD or PPA. Black: clinical diagnosis of ALS or PSMA. Grey: relatives of index patients affected by other forms of dementia or psychiatric disorders. Numbers inside symbols represent additional family members without further clinical information. Numbers below the symbols indicate age at death or current age. Clinical diagnoses: bvFTD, behavioural variant of frontotemporal dementia; svPPA, semantic variant of primary progressive aphasia; ALS, amyotrophiclateral sclerosis; PSMA, progressive spinal muscular atrophy; UD, unspecified dementia; Psych, psychiatric disorder; NA, not affected based on family history; unk, disease status unknown. *Neuropathological examination (patient 1F). Lower left panel: neuroimaging of three FTD patients with the I383V variant showing predominant bitemporal atrophy. MRI scans were obtained 7 years (1C), 6 years (1E) and 9 years (2H) after symptom onset. Quantitative analysis of volumetric loss per brain region is shown in online supplemental figure 2. Lower right panel: immunohistochemistry of patient 1F revealed several pTDP-43 positive neuronalcytoplasmic inclusions (NCI) of various morphologies in the frontal cortex (A) and nucleus caudate (B). Compared to other FTD-TDP cases, the amount of inclusions is low and intranuclear inclusions were not found. Therefore, this patient could not be readily classified into one of the FTLD-TDP subtypes. Staining with AT8 antibody revealed NCI in the hippocampus (C) and tufted astrocytes in nucleus caudate (D). Although this patient was 81 years at death, the observed tau pathology is not compatible with normal aging.

Six FTD patients and one ALS patient were found to be related (family 1). Additionally, two FTD patients and two ALS patients (families 2 and 3) could be linked to family 1 through a distant common ancestor (figure 1). The variable phenotype of the I383V variant is exemplified by family 1, in which different family members were affected by svPPA, behavioural variant of FTD, unspecified dementia, ALS or progressive spinal muscular atrophy, with a wide range in age at onset (44–69 years) and disease duration (7–23 years). Interestingly, several obligate carriers were unaffected, suggesting incomplete penetrance even at an advanced age (>80 years). Larger prospective studies are required to estimate age-related penetrance.

Four remaining families (online supplemental figure 1) did not show a clear pattern of autosomal dominant inheritance (Goldman 2–5). In one of these families, an affected relative with the I383V variant was clinically diagnosed with Alzheimer’s disease (AD), but AD biomarker changes were not evaluated in cerebrospinal fluid. A possible explanation is that the dementia in this patient is coincidental and unrelated to the I383V variant. Alternatively, increased susceptibility for AD caused by the I383V variant may be considered. Another interesting hypothesis is that *TARDBP* variants might be associated with limbic-predominant age-related TDP-43 encephalopathy, a common age-related disorder with TDP-43 proteinopathy that clinically mimics AD.^3^

10.1136/jnnp-2020-325150.supp3 Supplementary data

Several other relatives, including obligate carriers, were affected by psychiatric disorders such as psychosis and schizophrenia with onset around 40–50 years. Unfortunately, detailed clinical information or DNA were not available for these subjects. Whether psychiatric disorders are part of the I383V*–TARDBP* spectrum remains to be investigated in future studies. Altogether, our observations illustrate large phenotypic variability of the I383V variant and incomplete penetrance.

### Isolated bitemporal atrophy in FTD patients with the I383V variant

The most discriminating feature of the I383V variant is the predominant and severe atrophy of the temporal lobes in all FTD patients, with relative sparing of the other lobes (figure 1 and online supplemental figure 2). This is in line with previous observations in I383V FTD patients and the frequent occurrence of semantic deficits and prosopagnosia in our patients (online supplemental table 1). Other pathogenic *TARDBP* variants (eg, K263E) are associated with a more variable pattern of lobar atrophy.^1^ However, predominant temporal involvement has also been reported for other *TARDBP* variants located nearby the I383V variant (eg, A382T),^4^ suggesting a specific effect of missense variants in this part of the C-terminal domain of TDP-43. Further functional studies are needed to elucidate these possible genotype–phenotype correlations.

10.1136/jnnp-2020-325150.supp4 Supplementary data

### Heterogeneous pathological features in *TARDBP* patients

A remarkable observation is the scarcity of TDP-43 reactivity in the cortical areas of two FTD patients (patient 1F and the previously reported patient 4M^1^), despite the underlying pathogenic *TARDBP* variant. Only several TDP-43 cytoplasmic inclusions of various morphologies were found in the frontal cortex, dentate gyrus and caudate nucleus (figure 1). A possible explanation for the scarce temporal pathology might be the severe neurodegeneration, especially considering the long disease duration of patient 1F (23 years). Interestingly, we also detected tau positive inclusions in the hippocampus and tufted astrocytes in the putamen and caudate nucleus (figure 1). A single other neuropathological study of a I383V carrier reported similar low amounts of TDP-43 inclusions, and the presence of α-synuclein deposits and tauopathy, including tufted astrocytes in the amygdala.^5^ It appears that the neuropathological changes in FTD caused by variants in *TARDBP* are not readily classifiable. Whether the detected co-pathologies occurred by chance needs to be determined in additional cases with TDP-43 variants.

### Classification of the I383V variant as likely pathogenic

Our findings indicate a pathogenic effect of the I383V variant, which was previously debated due to the more conservative amino acid substitution and the benign in silico predictions by SIFT and PolyPhen. The current families, especially family 1, clearly show segregation of the variant with the disease, although penetrance appears incomplete. In addition to the patients described here, the I383V variant has been previously reported in 16 FTD and 8 ALS patients (online supplemental table 3), with frequencies ranging from 0% to 0.9% in ALS cohorts and from 0% to 2.5% in clinical FTD cohorts, while the variant is consistently absent in large groups of healthy controls from different populations. These data additionally support its pathogenicity. This conclusion has clinical implications for genetic counselling of patients and unaffected family members, to whom presymptomatic testing and counselling can now be offered.

## Conclusion

Our study provides sufficient evidence for the pathogenicity of the I383V variant and contributes to the characterisation of *TARDBP*-related FTD. We demonstrate the large phenotypic variability and incomplete penetrance of the I383V variant. Marked isolated bitemporal volume loss in all FTD patients should prompt clinicians to genetically test for causal variants in *TARDBP*.
