# Supplementary material for: Distinctive pattern of temporal atrophy in patients with frontotemporal dementia and the I383V variant in TARDBP
Source: J Neurol Neurosurg Psychiatry. 2021 Jan 15;92(7):787–9. doi: 10.1136/jnnp-2020-325150 (PMC8223666; doi:10.1136/jnnp-2020-325150)
Supplement: Supplementary data [file jnnp-2020-325150supp001.pdf]

SUPPLEMENTARY METHODS

Patient selection

We ascertained FTD patients (*n*=13) with the variant I383V (NM_007375.3: c.1147A>G, p.Ile383Val) in the *TARDBP* gene, from a large combined cohort of dementia patients who underwent clinical and genetic evaluation in two medical centers in the Netherlands (Amsterdam UMC, Vrije Universiteit Amsterdam, and Erasmus Medical Center, Rotterdam). All but one patient were included in either the Amsterdam Dementia Cohort[1] or an ongoing genetic-epidemiologic study of frontotemporal dementia.[2]

Subsequently, ALS patients (*n*=4) with the I383V variant in *TARDBP* were selected from the largest ALS cohort in the Netherlands (ALS Center, University Medical Center Utrecht), comprising over 4000 ALS patients included in Project MinE.[3] Cognitive status was evaluated in ALS patients on indication only.

Neurological examination and neuroimaging

All FTD patients included in this study underwent neurological and cognitive assessment, and routine neuroimaging (MRI or CT) as part of standard clinical practice. Clinical diagnoses were made according to international consensus criteria.[4-6] No neurophysiological assessment was performed. All imaging data were evaluated by experienced neuroradiologists. Additionally, volume loss across all lobar brain regions was quantitatively assessed in patients when 3D-acquired T1-weighted MRI scans with sufficient quality were available (*n*=5). Quantib® ND 1.6 software (Quantib, Rotterdam, The Netherlands), was used to generate automated segmentation and quantification of brain tissue. Volumes were compared to a gender-/age-matched reference population.

Genetic analyses – FTD patients

In all FTD patients, whole-exome sequencing (WES) or whole-genome sequencing (WGS) was performed in either clinical or research setting. Besides the I383V variant, concurrent pathogenic variants in 20 other genes associated with ALS, FTD or other forms of dementia were excluded (Table 1). The presence of a *C9orf72* repeat expansion was tested either using repeat-primed PCR (research setting) or a commercial kit (Asuragen® AmplideX PCR/CE; diagnostic setting) with repeat length ≥30 considered pathogenic.[7] The variant I383V in *TARDBP* was confirmed by Sanger sequencing.

In 11 out of 13 FTD patients, whole-exome sequencing was performed. DNA was enriched using Agilent SureSelect Clinical Research Exome V2 capture, fragmented to 150 to 200 base pairs, end paired, adenylated, and ligated to adapters. The SeqCap capturing kit for Illumina Paired-End Sequencing library (version 2.0.1; NimbleGen) was used. The captured fragments were purified, and sequenced on either an Illumina Hiseq2000 (Erasmus Medical Center) or Hiseq4000 platform (Amsterdam University Medical Center) using 100 bp paired-end reads. The aim was to obtain 8.1 Giga base pairs per exome with a mapped fraction of 0.99. The average coverage of the exome is ~50x with a minimum depth of >30 reads. Duplicate reads were excluded. Data were demultiplexed with bcl2fastq Conversion Software from Illumina. All sequence reads were mapped to GRCh37/hg19 reference genome using Burrows-Wheeler Aligner (BWA) Tool.[8] GATK was used for variant calling and quality control according to best practice (McKenna, et al., 2010). Population database frequencies (gnomAD v2.1.1), functional and impact-score annotations were assigned to variants using ANNOVAR.[9]

In 2 out of 13 FTD patients, whole-genome sequencing was performed as part of another study[10], at the Mayo Clinic Genome Analysis Core. Paired end libraries were prepared using 500ng of genomic DNA according to the manufacturer's instructions for the Nextera DNA Flex Library Prep

Kit (Illumina). Libraries were sequenced at an average coverage of ~30X (24 samples/S4 Flow cell) following Illumina's standard protocol using the Illumina NovaSeq™ 6000 and S4 flow cell. The flow cells were sequenced as 150X 2 paired end reads using NovaSeq S4 sequencing kit and NovaSeq Control Software v1.6.0. Base-calling is performed using Illumina's RTA version 3.4.4. Fastq files were processed through the Mayo Genome GPS v4.0 pipeline in a single batch of 48 samples. Briefly, reads were mapped to the human reference sequence (GRCh38 build) using the Burrows–Wheeler Aligner[8], and local realignment around indels was performed using the Genome Analysis Toolkit (GATK).[11] Variant calling was performed using GATK HaplotypeCaller followed by variant recalibration (VQSR) according to the GATK best practice recommendations. [12, 13] Joint genotyping including all samples was performed using GATK GenotypeGVCF. Quality control (QC) analysis of the data was conducted using a Mayo Clinic in house developed next generation sequencing (NGS) QC pipeline.

Genetic analyses – ALS patients

The four ALS patients described in this study were included in project MinE, a large-scale whole-genome sequencing study in ALS. For methodological details, we refer to previously published papers on the project MinE ALS sequencing consortium.[3, 14] A *C9orf72* repeat expansion was excluded in these patients by repeat-primed PCR[15] or using the WGS data and the software tool ExpansionHunter.[16]

Genealogical analysis

Family histories for FTLD spectrum disorders (bvFTD, PPA, ALS, CBS or PSP) were classified into one of the following Goldman categories, which were adjusted and described in more detail previously[10]:

1) Autosomal dominant pattern; 2) Familial aggregation; 3) Possible familial with onset <65 years; 4) Possible familial with onset >65 years; 5) Negative family history for a FTLD spectrum disorder, any other type of dementia, or Parkinson's disease (PD).

Psychiatric family history was assessed separately.

We performed genealogical research to trace a common link between the FTD patients and the ALS patients. The used sources included Dutch civil registries of births, marriages and deaths (1811-2020) and church archives with baptism, marriage, and death registers (before 1811).

Pathological examination

Brain autopsy was performed in two FTD patients by the Netherlands Brain Bank (NBB) within four hours after death. Routine immunohistochemistry was also carried out by the NBB and FTLD diagnosis was confirmed by a neuropathologist based on the criteria by Cairns et al.[17] We performed additional immunohistochemistry on multiple brain regions including all cortical areas, hippocampus and caudate/putamen as previously described.[2] One patient (4M) was reported previously as M008015-001.[18]

Symbol	Name	Reference
ANG	Angiogenin	Greenway et al., 2006
APP	Amyloid beta precursor protein	Goate et al., 1991
CHCHD10	Coiled-coil-helix-coiled-coil-helix domain containing 10	Claussenot et al., 2014
CHMP2B	Charged multivesicular body protein 2B	Skibinski et al., 2005
FUS	FUS RNA binding protein	Vance et al., 2009, Huey et al., 2012
GRN	Granulin Precursor	Cruts et al., 2006
HNRNPA1	Heterogeneous Nuclear Ribonucleoprotein A1	Kim et al., 2013
HNRNPA2B1	Heterogeneous nuclear ribonucleoprotein A2/B1	Kim et al., 2013
MAPT	Microtubule associated protein tau	Hutton et al., 1998
OPTN	Optineurin	Belzil et al., 2011; Pottier et al., 2018
PRKAR1B	Protein kinase cAMP-dependent type I regulatory subunit beta	Wong et al., 2014
PSEN1	Presenilin 1	Sherrington et al., 1995
PSEN2	Presenilin 2	Levy-Lahad et al., 1995
SIGMAR1	Sigma Non-Opioid Intracellular Receptor 1	Luty et al., 2010, Belzil et al., 2013
SOD1	Superoxide Dismutase 1	Rosen et al., 1993
SQSTM1	Sequestosome 1	Le Ber et al., 2013, Thelen et al., 2014
TARDBP	TAR DNA Binding Protein	Caroppo et al., 2016
TBK1	TANK binding kinase 1	Van der Zee et al., 2017
TREM2	Triggering Receptor Expressed On Myeloid Cells 2	Borroni et al., 2014
UBQLN2	Ubiquilin 2	Dillen et al., 2013
VCP	Valosin Containing Protein	Watts et al., 2004; Wong et al., 2018

Table 1. A total of 21 genes associated with FTD, ALS, FTD-ALS, and Alzheimer’s disease were screened for variants using whole-exome or whole-genome sequencing, which was performed in all patients.

References

1. van der Flier WM, Pijnenburg YA, Prins N, et al. Optimizing patient care and research: the Amsterdam Dementia Cohort. J Alzheimers Dis 2014;41:313-327.
2. Seelaar H, Kamphorst W, Rosso SM, et al. Distinct genetic forms of frontotemporal dementia. Neurology 2008;71:1220-1226.
3. Project Min EALSSC. Project MinE: study design and pilot analyses of a large-scale whole-genome sequencing study in amyotrophic lateral sclerosis. Eur J Hum Genet 2018;26:1537-1546.
4. Brooks BR, Miller RG, Swash M, Munsat TL, World Federation of Neurology Research Group on Motor Neuron D. El Escorial revisited: revised criteria for the diagnosis of amyotrophic lateral sclerosis. Amyotroph Lateral Scler Other Motor Neuron Disord 2000;1:293-299.
5. Gorno-Tempini ML, Dronkers NF, Rankin KP, et al. Cognition and anatomy in three variants of primary progressive aphasia. Ann Neurol 2004;55:335-346.
6. Rascovsky K, Hodges JR, Knopman D, et al. Sensitivity of revised diagnostic criteria for the behavioural variant of frontotemporal dementia. Brain 2011;134:2456-2477.
7. Renton AE, Majounie E, Waite A, et al. A hexanucleotide repeat expansion in C9ORF72 is the cause of chromosome 9p21-linked ALS-FTD. Neuron 2011;72:257-268.
8. Li H, Durbin R. Fast and accurate short read alignment with Burrows–Wheeler transform. bioinformatics 2009;25:1754-1760.
9. Wang K, Li M, Hakonarson H. ANNOVAR: functional annotation of genetic variants from high-throughput sequencing data. Nucleic acids research 2010;38:e164-e164.
10. Mol MO, van Rooij JGJ, Wong TH, et al. Underlying genetic variation in familial frontotemporal dementia: sequencing of 198 patients. Neurobiology of Aging 2020.
11. McKenna A, Hanna M, Banks E, et al. The Genome Analysis Toolkit: a MapReduce framework for analyzing next-generation DNA sequencing data. Genome Res 2010;20:1297-1303.
12. DePristo MA, Banks E, Poplin R, et al. A framework for variation discovery and genotyping using next-generation DNA sequencing data. Nat Genet 2011;43:491-498.
13. Van der Auwera GA, Carneiro MO, Hartl C, et al. From FastQ data to high confidence variant calls: the Genome Analysis Toolkit best practices pipeline. Curr Protoc Bioinformatics 2013;43:11 10 11-11 10 33.
14. van der Spek RAA, van Rheenen W, Pulit SL, et al. The project MinE databrowser: bringing large-scale whole-genome sequencing in ALS to researchers and the public. Amyotroph Lateral Scler Frontotemporal Degener 2019;20:432-440.
15. van Rheenen W, van Blitterswijk M, Huisman MH, et al. Hexanucleotide repeat expansions in C9ORF72 in the spectrum of motor neuron diseases. Neurology 2012;79:878-882.
16. Dolzhenko E, van Vugt J, Shaw RJ, et al. Detection of long repeat expansions from PCR-free whole-genome sequence data. Genome Res 2017;27:1895-1903.
17. Cairns NJ, Bigio EH, Mackenzie IR, et al. Neuropathologic diagnostic and nosologic criteria for frontotemporal lobar degeneration: consensus of the Consortium for Frontotemporal Lobar Degeneration. Acta Neuropathol 2007;114:5-22.
18. Caroppo P, Camuzat A, Guillot-Noel L, et al. Defining the spectrum of frontotemporal dementias associated with TARDBP mutations. Neurol Genet 2016;2:e80.
