# Supplementary material for: Distinctive pattern of temporal atrophy in patients with frontotemporal dementia and the I383V variant in TARDBP
Source: J Neurol Neurosurg Psychiatry. 2021 Jan 15;92(7):787–9. doi: 10.1136/jnnp-2020-325150 (PMC8223666; doi:10.1136/jnnp-2020-325150)
Supplement: Supplementary data [file jnnp-2020-325150supp002.pdf]

	1A	1C	1D	1E	1F[a]	1G	2H	3K	4L	4M	5O	6P	7Q
**Gender**	F	F	F	M	F	M	M	F	M	M	M	M	M
**Age at onset**	55	69	55	44	58	46	42	47	54	65	49	60	49
**Disease duration (years)**	8	10	2	10	23*	8*	11	11	5	7*	17*	8	12
**Clinical diagnosis**	bvFTD	bvFTD	bvFTD	bvFTD	bvFTD	svPPA	bvFTD	bvFTD	bvFTD	bvFTD	svPPA	bvFTD	bvFTD
**Clinical symptoms**													
Behavioral disinhibition	++	++	+	+	+	-	++	-	+	++	+	-	+
Apathy/loss of sympathy	+	+	-	+	±	+	++	+	±	±	++	+	-
Compulsivity/stereotypes	-	-	-	+	+	+	+	+	+	-	+	+	-
Hyperorality/dietary changes	+	-	-	-	+	-	-	-	++	-	++	-	-
Executive deficits	+	+	+	-	+	-	+	-	-	+	±	-	+
Episodic memory deficits	-	+	-	+	-	-	-	-	+	-	+	+	-
Impaired confrontation naming	+	++	±	+	+	++	+	++	+	-	+	+	+
Impaired language comprehension	+	+	+	-	±	++	+	-	-	-	+	±	-
Prosopagnosia	+	+	-	-	+	+	+	-	+	-	-	+	+
**Family history[b]**													
Goldman score	1	3	1	3	3	2	4	5	5	4	4	2	3
Psychiatric Family history	-	-	-	-	+	+	-	-	+	-	-	-	+
**Neuroimaging**													
Temporal atrophy at first scan	R>L (3)	R>L (5)	L>R (2)	L>R (5)	S (6)[c]	S (6)	S (8)	R>L (8)	R>L (4)	S (6)	R>L(5)	S (5)[c]	S (10)
Temporal atrophy at follow-up		S (7)		S (6)				R>L (11)					

Supplementary Table 1. Clinical characteristics of 13 FTD patients carrying I383V *TARDBP* variant.

Patients are numbered according to their ID in the pedigrees (Figure 1 / Supplementary Figure 1). Four out of 13 patients have died (disease duration marked with asterisk), whereas current disease duration is presented for the nine living patients. *Clinical diagnoses:* bvFTD = behavioral variant of FTD; svPPA = semantic variant of primary progressive aphasia. *Clinical symptoms*: - = absent; ± = mild; + = moderate; ++ = severe. *Neuroimaging*: R = right; L = left; S = symmetric. The numbers in brackets indicate the number of years after symptom onset when neuroimaging was performed.

[a] Neuropathologic examination.

[b] Goldman score based on family history (up till 2nd degree).

[c] Computed tomography (CT) was performed instead of Magnetic Resonance Imaging (MRI).

	1B	3I	3J	5N
**Gender**	M	F	F	M
**Age at onset**	39	53	59	62
**Disease duration (years)**	6*	6	9*	1*
**Clinical diagnosis**	ALS	PSMA	ALS	ALS
**Clinical symptoms**				
Onset	Spinal	Spinal	Spinal	Spinal
EMG affected regions (N)[a]	1	0[d]	2	3
Diagnostic delay (months)[b]	12	64	24	8
Cognitive impairment	-	-	-	-
**Family history**[c]				
Goldman score	1	5	5	5
Psychiatric Family history	-	-	-	-

Supplementary Table 2. Clinical characteristics of four ALS patients carrying I383V *TARDBP* variant.

Patients are numbered according to their ID in the pedigrees (Figure 1 / Supplementary Figure 1). Three patients have died (disease duration marked with asterisk), whereas current disease duration is presented for patient 3I. Neuroimaging was not performed in these patients. *Clinical diagnoses:* ALS = amyotrophic lateral sclerosis; PSMA = progressive spinal muscular atrophy.

[a] Number of regions with features consistent with ALS according to the revised El Escorial criteria.[1]

[b] The diagnostic delay is presented to indicate the relative slow progression in patients 3I and 3J.

[c] Goldman score based on family history (up till 2nd degree).

[d] Denervation and reinnervation changes were present, though not fulfilling El Escorial criteria.

Authors	Number of patients (families)	Age at onset	Clinical Diagnosis	Predominant atrophy distribution	Family history
Ramos et al., 2020[2]	4 (2)	ND	FTD	ND	Positive
Ramos et al., 2019[3]	3 (3)	60	bvFTD	Temporal (left>right)	Negative
		58	svPPA	Temporal (bilateral) + Frontal	Positive
		66	svPPA	Temporal (left>right) + Frontal	Positive
Gelpi et al., 2014[4]	1 (1)	60	svPPA	Temporal (left, bilateral at autopsy)	Dementia
Caroppo et al., 2016[5]	4 (3)	65	bvFTD-ALS	Temporal (bilateral) + Parietal (mild)	ALS
		63	bvFTD	Temporal (bilateral) + Frontal	bvFTD
		51	bvFTD	Temporal (right>left) + Frontal + Hippocampal	bvFTD
		51	svPPA	Temporal (left>right)	Negative
Cheng et al., 2016[6]	3 (1)	38	ALS	No atrophy	FTD, ALS
		64	svPPA	Temporal (left>right)	FTD, ALS
		62	bvFTD-ALS	Temporal (bilateral) + Frontal	FTD, ALS
Coyle-Gilchrist et al., 2016[7]	1 (1)	49	svPPA	Temporal (bilateral)	Unknown
Gonzalez-Sanchez et al., 2018[8]	2 (1)	51	svPPA	Temporal (left>right)	dementia, ALS
		52	ALS	ND	dementia, svPPA
Rutherford et al., 2008[9]	1 (1)	59	ALS	ND	ALS
Ticozzi et al., 2011[10]	3 (1)	66	ALS	ND	ALS
		25	ALS	ND	ALS
		57	ALS	ND	ALS
Ozoguz et al., 2015[11]	2 (1)	42	ALS	ND	ALS
		47	ALS	ND	ALS

Supplementary Table 3. Clinical characteristics of previously reported patients carrying the I383V *TARDBP* variant.
A total of 24 patients with the I383V variant from 17 different families have been reported in the literature. Eight of these patients were diagnosed with isolated ALS, whereas the majority was diagnosed with FTD or FTD-ALS. Neuroimaging of most FTD patients described the presence of temporal atrophy, in some cases combined with volume loss in other areas. *Clinical diagnoses:* ALS = amyotrophic lateral sclerosis; svPPA = semantic variant of primary progressive aphasia; bvFTD = behavioral variant of FTD. ND = not described.

References

1. Brooks BR, Miller RG, Swash M, Munsat TL, World Federation of Neurology Research Group on Motor Neuron D. El Escorial revisited: revised criteria for the diagnosis of amyotrophic lateral sclerosis. Amyotroph Lateral Scler Other Motor Neuron Disord 2000;1:293-299.
2. Ramos EM, Dokuru DR, Van Berlo V, et al. Genetic screening of a large series of North American sporadic and familial frontotemporal dementia cases. Alzheimers Dement 2020;16:118-130.
3. Ramos EM, Koros C, Dokuru DR, et al. Frontotemporal dementia spectrum: first genetic screen in a Greek cohort. Neurobiol Aging 2019;75:224 e221-224 e228.
4. Gelpi E, van der Zee J, Turon Estrada A, Van Broeckhoven C, Sanchez-Valle R. TARDBP mutation p.Ile383Val associated with semantic dementia and complex proteinopathy. Neuropathol Appl Neurobiol 2014;40:225-230.
5. Caroppo P, Camuzat A, Guillot-Noel L, et al. Defining the spectrum of frontotemporal dementias associated with TARDBP mutations. Neurol Genet 2016;2:e80.
6. Cheng YW, Lee MJ, Chen TF, et al. A single nucleotide TDP-43 mutation within a Taiwanese family: A multifaceted demon. Amyotroph Lateral Scler Frontotemporal Degener 2016;17:292-294.
7. Coyle-Gilchrist IT, Dick KM, Patterson K, et al. Prevalence, characteristics, and survival of frontotemporal lobar degeneration syndromes. Neurology 2016;86:1736-1743.
8. Gonzalez-Sanchez M, Puertas-Martin V, Esteban-Perez J, et al. TARDBP mutation associated with semantic variant primary progressive aphasia, case report and review of the literature. Neurocase 2018;24:301-305.
9. Rutherford NJ, Zhang YJ, Baker M, et al. Novel mutations in TARDBP (TDP-43) in patients with familial amyotrophic lateral sclerosis. PLoS Genet 2008;4:e1000193.
10. Ticozzi N, LeClerc AL, van Blitterswijk M, et al. Mutational analysis of TARDBP in neurodegenerative diseases. Neurobiol Aging 2011;32:2096-2099.
11. Ozoguz A, Uyan O, Birdal G, et al. The distinct genetic pattern of ALS in Turkey and novel mutations. Neurobiol Aging 2015;36:1764 e1769-1764 e1718.
