# Supplementary material for: Distinctive pattern of temporal atrophy in patients with frontotemporal dementia and the I383V variant in TARDBP
Source: J Neurol Neurosurg Psychiatry. 2021 Jan 15;92(7):787–9. doi: 10.1136/jnnp-2020-325150 (PMC8223666; doi:10.1136/jnnp-2020-325150)
Supplement: Supplementary data [file jnnp-2020-325150supp003.pdf]

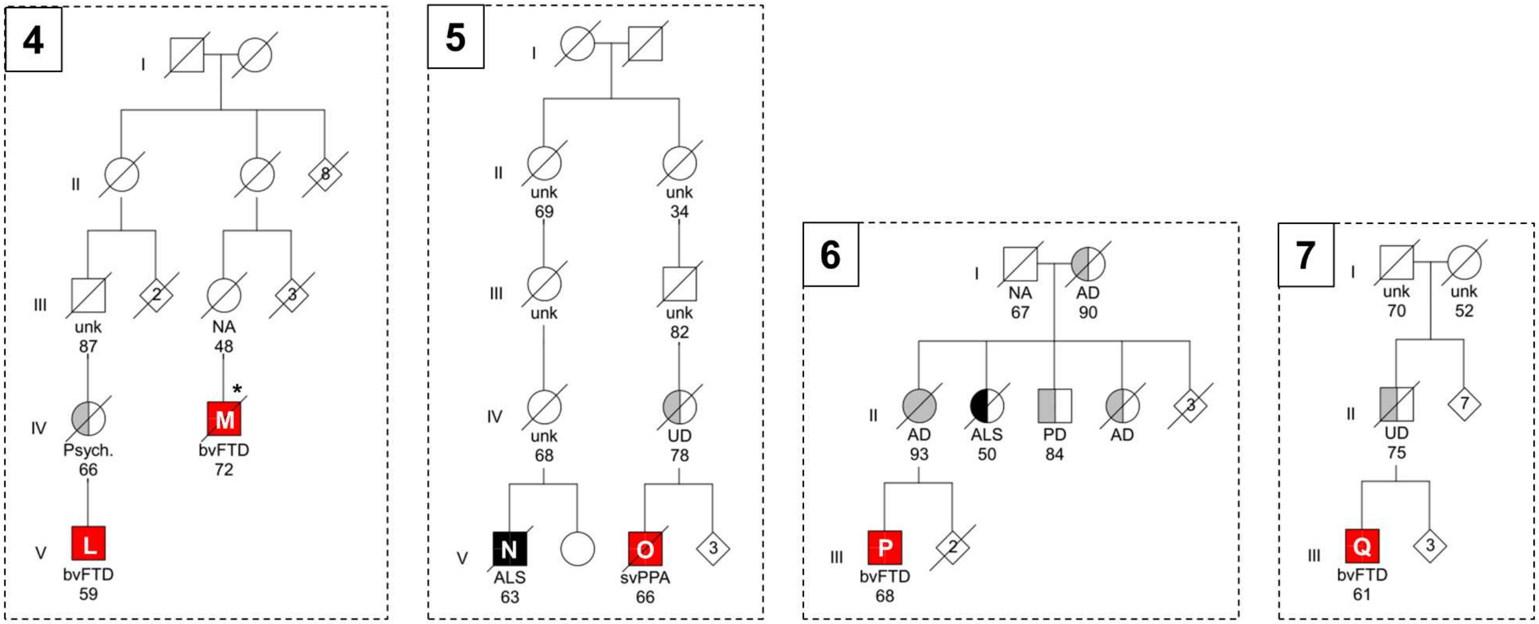

Supplementary Figure 1. Pedigrees of the families 4-7.

Four out of seven pedigrees are presented here, including five FTD patients and one ALS patient with a confirmed I383V variant in *TARDPB* (numbered L-Q). Fully colored symbols represent confirmed carriers of the I383V variant in *TARDBP* (*n*=7), including one relative clinically diagnosed with late-onset AD (family 6). Half colored symbols represent patients with a clinical diagnosis without genetic testing. *Red* = clinical diagnosis of FTD or PPA. *Black* = clinical diagnosis of ALS or PSMA. *Grey* = relatives of index patients affected by other forms of dementia or psychiatric disorder. Numbers inside symbols represent additional family members without further clinical information. Numbers below the symbols indicate age at death or current age.

Clinical diagnoses: bvFTD = behavioral variant of frontotemporal dementia; svPPA = semantic variant of primary progressive aphasia; ALS = amyotrophic lateral sclerosis; AD = Alzheimer's disease; PD = Parkinson's disease; UD = unspecified dementia; Psych. = psychiatric disorder; NA = not affected based on family history; unk = disease status unknown.

* Neuropathologic examination (patient 4M).
