# Supplementary material for: Distinctive pattern of temporal atrophy in patients with frontotemporal dementia and the I383V variant in TARDBP
Source: J Neurol Neurosurg Psychiatry. 2021 Jan 15;92(7):787–9. doi: 10.1136/jnnp-2020-325150 (PMC8223666; doi:10.1136/jnnp-2020-325150)
Supplement: Supplementary data [file jnnp-2020-325150supp004.pdf]

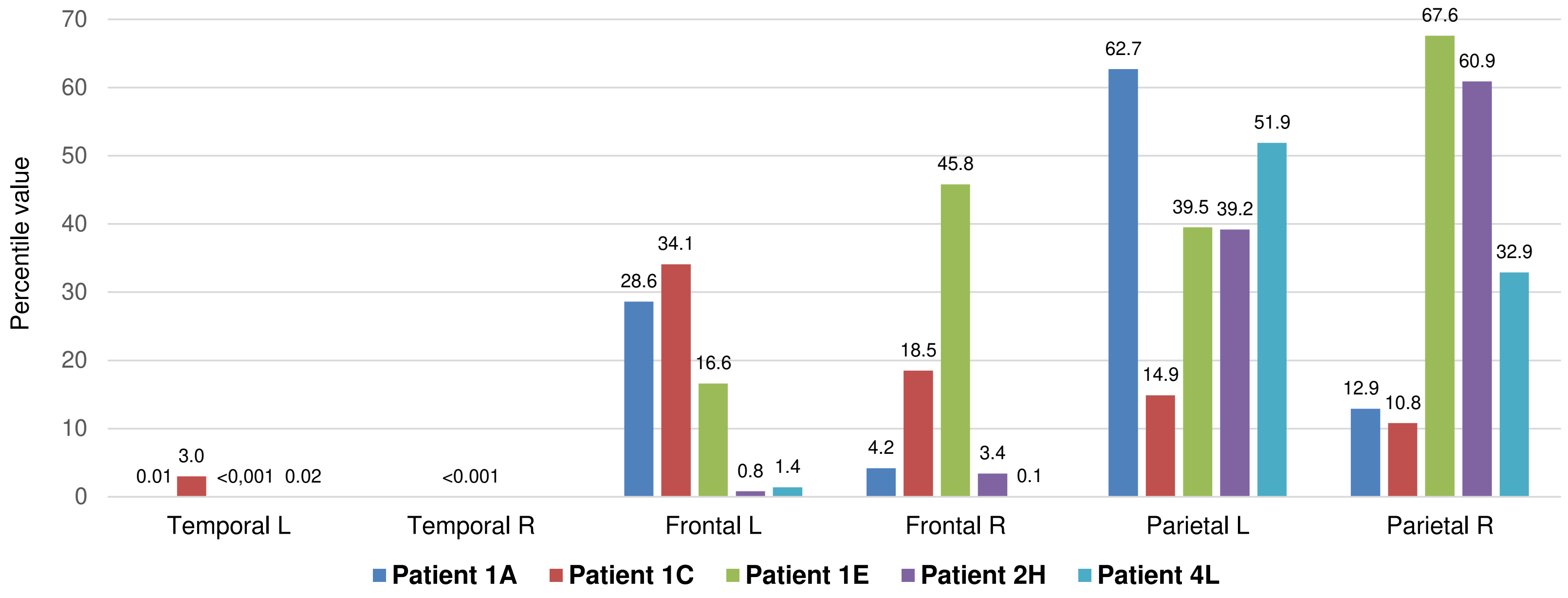

Supplementary Figure 2. Brain volumetric quantification of 5 FTD patients carrying the I383V *TARDBP* variant.

Patients are numbered according to their ID in the pedigrees (Figure 1 / Supplementary Figure 1). Volume loss across different brain regions was assessed in five patients using Quantib® ND 1.6 software and compared to a gender and age matched reference population, using so-called reference percentile curves. On the y-axis, age-specific and gender-specific percentile values are shown for left and right temporal, frontal, and parietal lobes. In general, percentile values <5% are considered abnormal.

Clearly visible is the marked bitemporal volume loss in all five patients. More subtle atrophy of the frontal lobes is present in three patients (1A, 2H, 4L). Asymmetry between the left and right temporal lobe, as visually observed, was not found with this method likely due to a floor effect. MRI scans were obtained 3 years (1A), 7 years (1C), 6 years (1E), 9 years (2H) and 4 years (4L) after symptom onset.
